# Census-tract broadband connectivity and the risk of diabetes, hypertension and obesity/overweight in California

**DOI:** 10.1186/s12889-025-25663-z

**Published:** 2025-11-27

**Authors:** Shane DeGrace, Norman Turk, Yasmine Alhoch, O. Kenrik Duru

**Affiliations:** 1 University of California San Diego School of Medicine, 9500 Gilman Drive La Jolla, San Diego, CA 92093 USA; 2 David Geffen School of Medicine, University of California, Los Angeles, 1100 Glendon Avenue, Suite 900, Los Angeles, CA 90024 USA; 3 UCLA Center for Health Policy Research, University of California, Los Angeles, 10960 Wilshire Blvd, Suite 1550, Los Angeles, CA 90024 USA

**Keywords:** Broadband, Internet, Diabetes, Hypertension, Obesity

## Abstract

**Background:**

In the digital age, a growing literature suggests that broadband coverage and high-speed internet access are social determinants of health. However, there is little research on regional broadband connectivity and its relationship to the prevalence of common health conditions such as type 2 diabetes, hypertension, and obesity/overweight. Using the California Health Interview Survey (CHIS) and US Census data, we aimed to assess if local broadband connectivity at the census tract level was related to prevalence of cardiometabolic dysfunction (diabetes, hypertension, and obesity/overweight) in these census tracts.

**Methods:**

Using a cross-sectional design, we analyzed data from 2019 California Health Interview Survey (CHIS) respondents aged 18 years or older (n = 21,905). Broadband connectivity and population density at the census tract level was determined from 2010 US Census data. Health information, secondary predictors, and covariates were self-reported by California Health Interview Survey CHIS respondents.

**Results:**

The prevalence of type 2 diabetes was greater in broadband connectivity areas with less than 40% high-speed connectivity (OR 2.4, 95% CI 1.6–3.7, p < 0.001) as compared with broadband connectivity areas with greater than 80% high-speed connectivity. As compared to areas with greater than 80% high-speed connectivity, we did not find differences in diabetes prevalence for areas with 40–60% high-speed connectivity (OR 1.2, 95% CI 0.8–1.7) or areas with 60–80% high-speed connectivity (OR 1.2, 95% CI 0.97–1.4). There were trends toward greater hypertension prevalence in areas with lower high-speed broadband connectivity as compared to higher broadband connectivity, but none of the differences reached statistical significance (all p values between 0.05 and 0.1). We did not find statistically differences in the prevalence of obesity/overweight for areas with different penetration of high-speed broadband connectivity.

**Conclusions:**

In California, the prevalence of cardiometabolic conditions, particularly type 2 diabetes, is highest in areas with lowest broadband connectivity. More research is needed to better understand the direct relationship between broadband connectivity, the usage of online health resources and the management or prevention of cardiometabolic dysfunction.

## Background

Diabetes, hypertension, and obesity or overweight are common chronic health conditions [1–3] that disproportionately affect individuals of low socioeconomic status [4–6] and people of color, including individuals with lower health literacy [7–10]. These three conditions are intertwined and each are associated with an increased risk of cardiovascular and cerebrovascular disease [11–13]. Moreover, all three of these cardiometabolic conditions are associated with significantly higher rates of mortality when compared to healthy individuals of the same age range [14, 15]. Almost 50% of California adult residents have either diabetes or prediabetes, while approximately 30% have hypertension [16, 17]. 

Patients with chronic conditions increasingly rely on online health resources to manage these conditions, accessing interactive websites designed to support patient self-efficacy, enhance self-management capability, connect in real-time with other patients in support groups, and ensure a reliable online interface when direct contact with a medical provider is infeasible or limited. Additionally, internet-based health resources can aid in decision making, help people take control of their health, and positively impact the doctor-patient relationship [18]. Of note, patients with higher rates of hypertension and type 2 diabetes tend to have lower health literacy, are less able to access health resources, and are significantly more likely to be African American or Hispanic [19–21]. 

The efficient use of interactive online resources depends on reliable broadband connectivity, to which not all Americans have equal access, particularly low-income households [22]. Higher quality internet access is generally associated with better health outcomes [23]. With the growing importance of online resources that was accelerated by the COVID-19 pandemic[24], it is important to understand the association between broadband connectivity, health outcomes, and related sociodemographic factors.

For this present study, we used representative data from the California Health Interview Survey (CHIS) to explore the aim of the study, which was to assess the cross-sectional association between local broadband connectivity and type 2 diabetes, hypertension, and obesity/overweight prevalence in California census tracts. Additionally, we assessed whether sociodemographic factors such as race, income, and population density were associated with potential access to online resources and these cardiometabolic conditions. We hypothesized that slower broadband speeds were significantly associated with higher rates of diabetes, hypertension, and obesity/overweight at the census tract level, using the threshold of *p* < 0.05.

## Methods

### Study data

This study used data from the Federal Communications Commission (FCC) Form 477 Internet Access Data published in 2019 to operationalize the level of broadband connectivity as a function of the number of internet connections per 1,000 households within a given Census tract. While the dataset represents all United States tracts, only the data from California Census tracts were used for this study. The tracts used in this dataset are defined in the 2010 census and were not redrawn until 2020.

The measures of broadband connectivity and population density were merged with the 2019 California Health Interview Survey (CHIS). This dataset is collected and maintained by the UCLA Center for Health Policy Research within the UCLA Fielding School of Public Health, using address-based sampling to obtain a cross-sectional, representative sample of the total civilian, noninstitutionalized Californian population. To date, it is the nation’s largest state health survey and a comprehensive source of data on Californians overall as well as on the state’s various racial and ethnic groups. This study used CHIS 2019 data, which sampled adults across 44 different county and county group strata, using an address-based sampling (ABS) methodology. Telephone and web interviews were conducted to collect data across a representative population of California residents, with an overall response rate of 12.2% (16.2% for the screener response rate and 75.2% for the extended interview response rate). Information on the sample design, survey content, and administrative protocols employed for CHIS data collection is available at the CHIS website [25]. This study was approved by the Institutional Review Board at the University of California, Los Angeles (IRB# 20–000583).

### Study sample

This study included non-institutionalized Californian adults aged 18 years and older (*n* = 21,905). The study sample was drawn from 8,057 California census tracts, representing all counties within California. Respondents were invited by CHIS to either complete the survey online or to be interviewed over the telephone by a member of the SQL Server Reporting Services (SSRS). The study sample included participants who self-identified as Hispanic (*n* = 3,976), Non-Hispanic White (*n* = 13,945), African American only (*n* = 823), American Indian/Alaska Native (*n* = 100), Asian (*n* = 2,518), Native Hawaiian/Pacific Islander (*n* = 48), or two or more races (*n* = 495).

### Outcome variables

The three primary outcomes were prevalence of (1) obesity or overweight, (2) having type 2 diabetes, and (3) having hypertension. Obesity/overweight was measured with post-survey calculation of body mass index (BMI) from self-reported height and weight. Survey respondents were either classified as underweight (BMI 0–18.49.49), normal weight (BMI 18.5–24.99.5.99), overweight (BMI 25–29.99.99) or obese (BMI 30+). Diabetes was measured with the survey question, “Other than pregnancy, has a doctor ever told you that you have diabetes or sugar diabetes” (yes/no). Following this question, participants were asked “Were you told that you had Type 1 or Type 2 diabetes?” A ‘yes’ was coded from participants responding with ‘Type 2.’ A ‘no’ was coded from participants responding with ‘inapplicable’ or ‘borderline or pre-diabetes.’ Participants who responded that they had ‘Type 1,’ ‘Another Type,’ ‘double diabetes,’ and ‘provider did not specify or unknown’ were excluded from the analysis examining diabetes as a primary outcome. Hypertension was measured with the survey question, “Has a doctor ever told you that you have high blood pressure” (yes/no). No was coded from participants responding with ‘No’ or ‘borderline hypertension.’

### Primary predictor variable

The primary predictor was broadband connectivity, defined as the percent of internet connections per 1,000 households of at least 10 megabits per second (Mbps) downstream/1 Mbps upstream within a census tract. Speed parameters were set based on prior research standardizing quality markers in which the US Census Bureau and FCC reported broadband connectivity data [24]. Our primary predictor was assigned to each individual based on the census tract within which they were residing, thus allowing us to conduct individual-level analyses based on census tract characteristics. Broadband connectivity was measured as a categorical variable with four categories: Less than 40% connectivity, 40–60% connectivity, 60–80% connectivity, or greater than 80% connectivity.

### Secondary predictors and covariates

The study used a range of sociodemographic and socioeconomic secondary predictors. Race and ethnicity were assessed to examine associated differences in both health outcomes and broadband connectivity, utilizing the racial categories: Hispanic, Non-Hispanic White, African American only, American Indian/Alaska Native only, Asian only, Native Hawaiian/Pacific Islander, and two or more races. Non-Hispanic White served as the reference category. We also assessed household income, and per California state-wide income reports before tax[26], we operationalized this variable using 5 income categories: less than $40,000, between $40,000 and $60,000, between $60,001 and $80,000, between $80,001 and $100,000, and the reference group of greater than $100,000. Finally, we assessed population density to examine differences in health outcomes and broadband connectivity. CHIS includes four categories of population density – urban, suburban, “second city,” and rural. We collapsed the first three categories to make comparisons between urban and rural census blocks, with urban serving as the reference category. All models also controlled for gender as well as a continuous measure of age.

### Statistical analyses

To produce population estimates from CHIS data, weights were applied to the sample data to compensate for the probability of selection and a variety of other factors, some directly resulting from the design and administration of the survey. The sample was weighted to represent the noninstitutionalized population for each sampling stratum and statewide. For these analyses, we first examined the data descriptively using unweighted frequencies and weighted percentages or weighted means and standard errors. We then constructed three separate multivariate logistic regression models to analyze the relationships between our primary predictor, outcomes, and secondary predictors (Statistical Analysis System (SAS) version 14.2).

## Results

As shown in Table 1, our analytic sample (*N* = 21,905) was majority female (55.8%), primarily from urban communities (89.8%), and was majority non-Hispanic (NH) White (63.5%). The average age was approximately 60 years, and 10.2% of participants had type 2 diabetes, 33.3% had hypertension, and 60.5% were overweight or obese. Most of our sample fell within income bands either making less than $40k a year (25.6%) or more than $100k a year (38.6%). Most of our sample also lived in census tracts in which 60% or more of the tract had at least 10Mbps downstream/1Mbps upstream broadband connectivity.

**Table 1:** Descriptive statistics of sample population

	Total Sample	Diabetes Sample	Hypertension Sample	Obesity/Overweight Sample
	*N* = 21,905	*N* = 2,242	*N* = 7,297	*N* = 13,256
Mean Age (SD)	59.9 (17.2)	66.1 (12.5)	66.1 (13.4)	57.6 (16.2)
Female (*n* = 12,223)	55.8%	63.6%	51.5%	50.4%
Population Density				
Urban (*n* = 19,675)	89.8%	90.8%	88.8%	89.4%
Rural (*n* = 2,230)	10.2%	9.2%	11.2%	10.6%
Race/Ethnicity				
Hispanic (*n* = 3,976)	18.3%	20.1%	14.6%	21.7%
Black, Non-Hispanic (*n* = 823)	3.8%	2.8%	5.3%	4.6%
Native American (*n* = 100)	0.5%	0.3%	0.6%	0.6%
Asian (*n* = 2,518)	11.5%	13.9%	9.1%	7.4%
Hawaiian/Pacific Islander (*n* = 48)	0.2%	0.3%	0.3%	0.2%
Multiracial (*n* = 495)	2.3%	2.6%	1.8%	2.4%
White, Non-Hispanic (*n* = 13,945)	63.5%	60.1%	68.3%	63.1%
Income				
Less than $40k (*n* = 5,577)	25.6%	24.2%	32.4%	27.2%
$40k − 60k (*n* = 2,830)	12.9%	10.9%	14.5%	13.6%
$60k- $80k (*n* = 2,817)	12.9%	11.7%	13.8%	13.0%
$80k − 100k (*n* = 2,197)	10.0%	10.0%	9.6%	10.3%
$100k or more (*n* = 8,484)	38.6%	43.2%	29.7%	35.9%
High Speed Broadband				
Less than 40% (*n* = 661)	3.0%	2.8%	3.6%	3.2%
40–60% (*n* = 1,806)	8.3%	7.0%	8.8%	9.1%
60–80% (*n* = 6,022)	27.6%	27.0%	29.0%	29.6%
80% or more (*n* = 13,416)	61.1%	63.2%	58.6%	58.1%

Table 2 shows the adjusted model of association between diabetes prevalence and regional broadband connectivity. The prevalence of diabetes was greater in broadband connectivity areas with less than 40% high-speed connectivity (OR 2.4, 95% CI 1.6–3.7, *p* < 0.001) as compared with broadband connectivity areas with greater than 80% high-speed connectivity). No significant difference in diabetes prevalence was found when comparing areas with 40–60% high-speed connectivity and 80% high-speed connectivity (OR 1.2, 0.8–1.6), or when comparing areas with 60–80% high-speed connectivity (OR 1.2, 95% CI 0.97–1.4) and 80% high-speed connectivity. Diabetes prevalence was greatest among individuals with income <$40,000 as compared to higher income bands, and was also greatest among Asian, Hispanic, African American, Hawaiian/Pacific Islander and multiracial individuals as compared to non-Hispanic Whites (Table 2).

**Table 2:** Multivariate results examining broadband connectivity and odds of diabetes

	Odds Ratio for Diabetes	95% Confidence Limits	*p*-value
Census Tract Broadband Connectivity			
Less than 40%	2.4	1.6–3.7	< 0.001
40–60%	1.2	0.8–1.6	0.43
60–80%	1.2	0.97–1.4	0.10
80% or more (ref)	-	-	-
Covariates			
Race/Ethnicity			
Hispanic	2.5	2.0–3.0	< 0.001
African American, Non-Hispanic	2.1	1.5–2.9	< 0.001
Native American	0.75	0.3–1.7	0.48
Asian	2.2	1.8–2.7	< 0.001
Hawaiian/Pacific Islander	3.0	1.3–6.8	0.01
Multiracial	1.9	1.2–3.1	0.01
White, Non-Hispanic (ref)	-	-	-
Income			
Less than $40k	1.7	1.4–2.1	< 0.001
$40k − 60k	1.3	1.01–1.6	0.04
$60k- $80k	1.2	0.9–1.6	0.30
$80k − 100k	1.4	1.1–1.78	0.02
$100k or more (ref)	-	-	-
Population Density			
Urban	0.9	0.6–1.2	0.35
Rural (ref)	-	-	-

Multivariate logistic regression adjusted for the above covariates: race/ethnicity, income, urban vs. rural status

As shown in Table 3, there were trends toward greater hypertension prevalence in areas with lower high-speed broadband connectivity as compared to higher broadband connectivity, but none of the differences reached statistical significance (all p values between 0.05 and 0.1). Like our findings regarding diabetes, the prevalence of hypertension was highest among individuals with income <$40,000, and also highest among several racial and ethnic minority groups (Hispanic, African American, Hawaiian/Pacific Islander and Native American) as compared to non-Hispanic White individuals (Table 3).

**Table 3:** Multivariate results examining broadband connectivity and odds of hypertension

	Odds Ratio for Hypertension	95% Confidence Limits	*p*-value
Census Tract Broadband Connectivity			
Less than 40%	1.4	0.98–2.0	0.07
40–60%	1.3	0.99–1.6	0.06
60–80%	1.1	0.98–1.3	0.09
80% or more (ref)	-	-	-
Covariates			
Race/Ethnicity			
Hispanic	1.2	1.02–1.4	0.02
Black, Non-Hispanic	1.8	1.4–2.3	< 0.001
Native American	1.4	0.7–2.9	0.37
Asian	1.04	0.9–1.2	0.63
Hawaiian/Pacific Islander	2.9	1.5–5.4	0.001
Multiracial	0.8	0.6–1.2	0.34
White, Non-Hispanic	-	-	-
Income			
Less than $40k	1.9	1.6–2.1	< 0.001
$40k − 60k	1.3	1.2–1.6	< 0.001
$60k- $80k	1.2	0.97–1.5	0.09
$80k − 100k	1.2	0.98–1.4	0.08
$100k or more (ref)	-	-	-
Population Density			
Urban	0.98	0.8–1.2	0.88
Rural (ref)	-	-	-

Multivariate logistic regression adjusted for the above covariates: race/ethnicity, income, urban vs. rural status

As shown in Table 4, we did not find statistically differences in the prevalence of obesity/overweight for areas with different penetration of high-speed broadband connectivity. Rates of overweight/obesity were higher among both individuals with incomes <$40,000 (OR 1.2, 95% CI 1.1–1.4, *p* = 0.001) and between $40,000 and $60,000 (OR 1.2, 95% CI 1.1–1.5) as compared to those with incomes >$100,000. Rates of overweight/obesity were higher among Hispanic, African American, and Native American individuals as compared to non-Hispanic White individuals (Table 4). However, rates of overweight/obesity were lower among Asian individuals (OR 0.5, 95% CI 0.4–0.6, *p* < 0.001) as compared with non-Hispanic White individuals. Finally, overweight/obesity rates were higher among individuals in urban areas (OR 1.2, 95% CI 1.02–1.5, *p* = 0.028) as compared with individuals in rural areas.

**Table 4:** Multivariate results examining broadband connectivity and odds of Obesity/Overweight

	Odds Ratio for Obesity/Overweight	95% Confidence Limits	*p*-value
Census Tract Broadband Connectivity			
Less than 40%	1.1	0.8–1.4	0.74
40–60%	1.1	0.9–1.4	0.35
60–80%	1.03	0.9–1.2	0.61
80% or more (ref)	-	-	-
Covariates			
Race/Ethnicity			
Hispanic	2.1	1.8–2.4	< 0.001
Black, Non-Hispanic	1.3	1.2–1.8	0.002
Native American	5.4	2.3–12.5	< 0.001
Asian	0.5	0.4–0.6	< 0.001
Hawaiian/Pacific Islander	0.9	0.5–1.6	0.73
Multiracial	1.2	0.9–1.6	0.20
White, Non-Hispanic	-	-	-
Income			
Less than $40k	1.2	1.1–1.4	0.001
$40k − 60k	1.2	1.1–1.5	0.01
$60k- $80k	1.1	0.9–1.2	0.39
$80k − 100k	1.2	1.01–1.4	0.04
$100k or more (ref)	-	-	-
Population Density			
Urban	1.2	1.02–1.45	0.03
Rural (ref)	-	-	-

Multivariate logistic regression adjusted for the above covariates: race/ethnicity, income, urban vs. rural status

## Discussion

In a population-based, representative dataset of Californians, we found a significant and independent association between lower neighborhood broadband connectivity speed and greater diabetes prevalence, with a non-significant trend of an association between lower neighborhood broadband access speed and greater hypertension prevalence. Given that online resources for disease self-management are playing an increasingly large role in clinical care, these results suggest the possibility of a regional “digital divide” in California associated with health outcomes. This is particularly relevant in the aftermath of the COVID-19 pandemic, since many interactions between patients and health professionals which were traditionally face-to-face are now increasingly over virtual internet connections that depend on adequate broadband connectivity [27]. Research has found that those with less reliable broadband access, in addition to other factors such as lower health and digital literacy, face significant barriers to accessing telehealth [28]. 

Patients with diabetes who have lower broadband speeds may be less able to access and benefit from high quality care [29]. As an example, people with diabetes who used an online diabetes management system implemented by the Palo Alto Medical Foundation saw a significant reduction in their A1C at 6 months in comparison to those in the control group, although these differences were not sustained at 12 months [30]. There are numerous other mobile applications and internet portals designed to encourage diabetes self-management education [31]. There is also growing evidence that these types of internet-based health resources are effective in aiding decision making, helping people take control of their health, and positively impacting the patient-doctor relationship [18]. The internet also serves as a mechanism for individuals with chronic conditions to connect with others who have similar conditions. One study found that diabetes self-management in participants who used a Diabetes Online Community Platform was positively impacted by engaging in supportive online conversations with other persons with diabetes, families, and healthcare providers [32]. 

Support for diabetes prevention is also increasingly provided online, so although we could not directly measure prediabetes in these analyses it is possible that lower broadband speeds in some neighborhoods could be associated with greater progression along the continuum of glycemic control from prediabetes to diabetes. About 38% of all US adults have prediabetes, and evidence-based strategies such as the Diabetes Prevention Program can reduce the risks of incident diabetes in this population [33]. Organizations such as the American Diabetes Association and the Centers for Disease Control and Prevention have developed interactive online modules, virtual handouts, and chat lines to educate and help individuals become more empowered in preventing diabetes [34, 35]. With limited broadband connectivity, individuals at risk may be less likely to access these prevention resources.

As with diabetes, hypertension is highly prevalent in the United States and often uncontrolled [36]. Effective hypertension management can be aided by self-measured blood pressure monitoring (SMBP), an evidence-based strategy for lowering blood pressure when combined with clinical support [37, 38]. However, the processing and collection of blood pressure data from these devices is limited by broadband access and speed [39]. 

Racial and ethnic minorities in our analyses, notably Black and Hispanic Californians, as well individuals with lower incomes, were significantly more likely than non-Hispanic White or higher income individuals to have diabetes or hypertension, and these disparities may be correlated with internet speed. Areas with lower rates of broadband access tend to also have higher rates of preventable hospitalizations and worse access to primary care physicians[23], which are related to the disparities identified by the current study in the prevalence of diabetes and hypertension. This could pose a future barrier to the health management of minority, low-income, and elderly populations, all of whom tend to have less access – or ability to properly use – telehealth, virtual visits, and online health resources [40–42]. 

As broadband connectivity likely has a strong correlation with other neighborhood and environmental variables that increase the risk of cardiometabolic conditions, it is challenging to determine causal inference. Unmeasured confounders such as air pollution, the availability of neighborhood green space, or high density of fast-food restaurants may explain some of all of the observed association between low broadband speed and diabetes prevalence. Other economic variables that are closely tied to broadband speed, such as the creation of new businesses and the growth of existing businesses may lead to more and higher-paying local jobs, which has been linked to lower rates of diabetes [43]. A recent task force report from the Federal Communications Commission showed similar findings to our analyses, with decreasing diabetes prevalence with increasing quintiles of broadband connectivity [23]. 

If future quasi-experimental studies support a causal link between lower broadband speeds and higher diabetes prevalence, then increasing high-speed broadband connectivity in both rural and urban communities may provide health as well as economic benefits. As part of the Broadband Equity Access and Deployment (BEAD) program, the Biden-Harris Administration has authorized a $900 million program to install over 12,000 miles of high-speed broadband across over 350 US counties [44]. Moreover, $475 million was granted for devices such as laptops and tablets to be distributed to qualifying Americans as a part of the Connected Device Grant Program. Another $100 million was devoted to outreach efforts about various broadband affordability programs through the Federal Communications Commission [45]. Efforts to increase awareness of and enrollment in these new infrastructure programs may support chronic disease prevention and self-management, with potential greater impact among low-income and minority individuals. Subsidies to helped to boost broadband use but are vulnerable to budget cuts. The Affordable Connectivity Program (ACP) was instituted during the COVID-19 pandemic, ensuring that eligible lower-income households had access to high-speed internet connections from any of 20 leading internet providers for under $30 per month [46]. While this legislation was not renewed by Congress and lapsed in June 2024, fourteen Internet Service Providers have agreed to keep the subsidized rates in place until the end of calendar year 2024 [47, 48]. Unfortunately, the long-term future of affordable broadband is up in the air for many Americans with chronic conditions who live in rural areas.

Our study has a few limitations. First, these analyses are cross sectional, so causal inferences between broadband connectivity and cardiometabolic dysfunction cannot be made. In addition, there is unmeasured confounding due to variables not included in the CHIS data. For example, internet literacy, number of devices per household, the use of smartphones to access the internet, and intergenerational household status are all possible confounders that have been linked to the frequency of internet access or the successful navigation of internet-based platforms [49, 50]. We also were unable to determine the duration of local residency of CHIS respondents and the timing of self-reported diagnoses, which are also likely to underestimate the true prevalence of these conditions. Moreover, our measure of broadband connectivity was area-based, and we did not have data on the individual household internet speeds of the CHIS study respondents. As with most modern telephone surveys, the low response rate raised the possibility of non-response bias. Finally, this study was limited to California survey participants, suggesting that further research is necessary to understand how infrastructural and demographic differences across the United States influence the relationship between broadband connectivity and cardiometabolic dysfunction.

## Conclusion

In summary, we found that California census tracts with lower broadband connectivity were significantly associated with higher prevalence of cardiometabolic dysfunction, particularly diabetes. To date, few prior studies have investigated whether broadband connectivity is related to cardiometabolic dysfunction including diabetes and hypertension. The current study emphasizes the importance of focusing future research with stronger study designs that can advance the understanding how broadband connectivity, and the subsequent usage of online health resources, may play a role in managing and preventing cardiometabolic dysfunction and associated health outcomes in the growing digital age.

## Data Availability

The data that support the findings of this study are available from California Health Interview Survey (CHIS) and US Census.

## Abbreviations

ABS: Address-based sampling
ACP: Affordable Connectivity Program
BEAD: Broadband Equity Access and Deployment
BMI: Body Mass Index
CHIS: California Health Interview Survey
CI: Confidence Intervals
COVID-19: Coronavirus Disease 2019
FCC: Federal Communications Commission
Mbps: Megabits per second
NH: Non-Hispanic
SAS: Statistical Analysis System
SMBP: Self-measured blood pressure monitoring
SSRS: SQL Server Reporting Services

## References

[1] Ostchega Y, Fryar CD, Nwankwo T, Nguyen DT. Hypertension prevalence among adults aged 18 and over: united States, 2017–2018. NCHS Data Brief. 2020;364:1–8. 32487290

[2] Centers for Disease Control and Prevention. National Diabetes Statistics Report. Available at: https://www.cdc.gov/diabetes/data/statistics-report/index.html. Accessed 20 Nov 2023.

[3] National Institute of Diabetes, Digestive and Kidney Diseases. Overweight & Obesity Statistics. Available at: https://www.niddk.nih.gov/health-information/health-statistics/overweight-obesity. Accessed 20 November 2023.

[4] Brown AF, Ettner SL, Piette J, et al. Socioeconomic position and health among persons with diabetes mellitus: a conceptual framework and review of the literature. Epidemiol Rev. 2004;26:63–77. 15234948 10.1093/epirev/mxh002

[5] Mohammed SH, Habtewold TD, Birhanu MM, et al. Neighbourhood socioeconomic status and overweight/obesity: a systematic review and meta-analysis of epidemiological studies. BMJ Open. 2019;9:e028238. 31727643 10.1136/bmjopen-2018-028238 PMC6886990

[6] Leng B, Jin Y, Li G, Chen L, Jin N. Socioeconomic position and health among persons with diabetes mellitus: a conceptual framework and review of the literature. Epidemiol Rev. 2004;26:63–77. 15234948 10.1093/epirev/mxh002

[7] Price JH, Khubchandani J, McKinney M, Braun R. Racial/ethnic disparities in chronic diseases of youths and access to health care in the United States. Biomed Res Int. 2013;2013:787616. 24175301 10.1155/2013/787616 PMC3794652

[8] Asharani PV, Lau JH, Roystonn K, et al. Health literacy and Diabetes knowledge: A Nationwide Survey in a Multi-Ethnic Population. Int J Environ Res Public Health. 2021;18:9316. 10.3390/ijerph18179316 PMC8431510 34501905

[9] Mohd Isa D, Shahar S, He FJ, Majid HA. Associations of health literacy with blood pressure and dietary salt intake among adults: a systematic review. Nutrients. 2021;13:4534. 34960086 10.3390/nu13124534 PMC8707038

[10] Michou M, Panagiotakos DB, Costarelli V. Low health literacy and excess body weight: a systematic review. Cent Eur J Public Health. 2018;26:234–41. 30419628 10.21101/cejph.a5172

[11] Powell-Wiley TM, Poirier P, Burke LE, American Heart Association Council on Lifestyle and Cardiometabolic Health; Council on Cardiovascular and Stroke Nursing; Council on Clinical Cardiology; Council on Epidemiology and Prevention; and Stroke Council, et al. Obesity and cardiovascular disease: a scientific statement from the American heart association. Circulation. 2021;143:e984-1010. 33882682 10.1161/CIR.0000000000000973 PMC8493650

[12] Feigin VL, Norrving B, Mensah GA. Global burden of stroke. Circ Res. 2017;120:439–48. 28154096 10.1161/CIRCRESAHA.116.308413

[13] Tackling G, Borhade MB. Hypertensive Heart Disease. In: StatPearls. Treasure Island (FL): StatPearls Publishing; 2023 Jan-. Available at: https://www.ncbi.nlm.nih.gov/books/NBK539800/. Accessed 23 November 2023. 30969622

[14] Di Angelantonio Emanuele, Bhupathiraju Shilpa N, et al. Body-mass index and all-cause mortality: individual-participant-data meta-analysis of 239 prospective studies in four continents. Lancet. 2016;388:776–86. 27423262 10.1016/S0140-6736(16)30175-1 PMC4995441

[15] Raghavan S, Vassy JL, Ho YL, et al. Diabetes mellitus-related all-cause and cardiovascular mortality in a national cohort of adults. J Am Heart Assoc. 2019;8:e011295. 30776949 10.1161/JAHA.118.011295 PMC6405678

[16] Babey SH, Wolstein J, Diamant AL, Goldstein H. Prediabetes in california: nearly half of California adults on path to diabetes. Policy Brief UCLA Cent Health Policy Res. 2016;(PB2016-1):1–8. 27197309

[17] Santo L, Okeyode T, National Ambulatory Medical Care Survey. : 2018 National Summary Tables. Available from: https://www.cdc.gov/nchs/data/ahcd/namcs_summary/2018-namcs-web-tables-508.pdf. Accessed 30 August 2024.

[18] Bussey LG, Sillence E. The role of internet resources in health decision-making: a qualitative study. Digit Health. 2019;7:2055207619888073. 10.1177/2055207619888073 PMC6843735 31741741

[19] Tajdar D, Lühmann D, Fertmann R, et al. Low health literacy is associated with higher risk of type 2 diabetes: a cross-sectional study in Germany. BMC Public Health. 2021;21:510. 33726714 10.1186/s12889-021-10508-2 PMC7962353

[20] Rodríguez JE, Campbell KM. Racial and ethnic disparities in prevalence and care of patients with type 2 diabetes. Clin Diabetes. 2017;35:66–70. 28144049 10.2337/cd15-0048 PMC5241767

[21] Zhang X, Bullard KM, Gregg EW, et al. Access to health care and control of ABCs of diabetes. Diabetes Care. 2012;35:1566–71. 22522664 10.2337/dc12-0081 PMC3379598

[22] Swenson K, Ghertner R. People in Low-Income households have less access to internet services. Office of the Assistant Secretary for Planning & Evaluation, U.S. Department of Health & Human Services. 2020.

[23] Federal Communications Commission. Mapping Broadband Health in America. 2017. Available at: https://www.fcc.gov/sites/default/files/connect2health.key_findings.pdf. Accessed 30 August 2024.

[24] Vogels EA, Perrin A, Rainie L, Anderson M. 53% of Americans say the internet has been essential during the COVID-19 outbreak. Pew Research Center. 2020.

[25] California Health Interview Survey. Available at: https://healthpolicy.ucla.edu/our-work/california-health-interview-survey-chis/chis-design-and-methods. Accessed 20 Nov 2023.

[26] U.S. Census Bureau. New statistics available from the 2016–2020 American Community Survey 5-year estimates. 2022. Retrieved from: https://www.census.gov/newsroom/press-kits/2021/acs-5-year.html.

[27] Koonin LM, Hoots B, Tsang CA, et al. Trends in the use of telehealth during the emergence of the COVID-19 pandemic - United States, January-March 2020. MMWR Morb Mortal Wkly Rep. 2020;69:1595–9. 33119561 10.15585/mmwr.mm6943a3 PMC7641006

[28] Pandit AA, Mahashabde RV, Brown CC, et al. Association between broadband capacity and telehealth utilization among medicare fee-for-service beneficiaries during the COVID-19 pandemic. J Telemed Telecare. 2023;1357633X:231166026. 10.1177/1357633X231166026 PMC10076155 37016902

[29] O’Shea AMJ, Baum A, Haraldsson B, et al. Association of adequacy of broadband internet service with access to primary care in the veterans health administration before and during the COVID-19 pandemic. JAMA Netw Open. 2022;5:e2236524. 36251295 10.1001/jamanetworkopen.2022.36524 PMC9577674

[30] Tang PC, Overhage JM, Chan AS, et al. Online disease management of diabetes: engaging and motivating patients online with enhanced resources-diabetes (EMPOWER-D), a randomized controlled trial. J Am Med Inform Assoc. 2013;20:526–34. 23171659 10.1136/amiajnl-2012-001263 PMC3628059

[31] Shah VN, Garg SK. Managing diabetes in the digital age. Clin Diabetes Endocrinol. 2015;1:16. 28702234 10.1186/s40842-015-0016-2 PMC5471958

[32] Litchman ML, Edelman LS, Donaldson GW. Effect of diabetes online community engagement on health indicators: cross-sectional study. JMIR Diabetes. 2018;3:e8. 30291079 10.2196/diabetes.8603 PMC6238850

[33] Knowler WC, Barrett-Connor E, Fowler SE, et al. Reduction in the incidence of type 2 diabetes with lifestyle intervention or Metformin. N Engl J Med. 2002;346:393–403. 11832527 10.1056/NEJMoa012512 PMC1370926

[34] American Diabetes Association. Diabetes Support Resources. Available at: https://professional.diabetes.org/content-page/diabetes-support-resources. Accessed 20 Nov 2023.

[35] Division of Diabetes Translation, Centers for Disease Control and Prevention. Resources and Publications. Available at: https://www.cdc.gov/diabetes/resources-publications/index.html. Accessed 20 Nov 2023.

[36] Chobufo MD, Gayam V, Soluny J, et al. Prevalence and control rates of hypertension in the USA: 2017–2018. Int J Cardiol Hypertens. 2020;6:100044. 33447770 10.1016/j.ijchy.2020.100044 PMC7803011

[37] Uhlig K, Patel K, Ip S, Kitsios GD, Balk EM. Self-measured blood pressure monitoring in the management of hypertension: a systematic review and meta-analysis. Ann Intern Med. 2013;159:185–94. 23922064 10.7326/0003-4819-159-3-201308060-00008

[38] Tucker KL, Sheppard JP, Stevens R, et al. Self-monitoring of blood pressure in hypertension: a systematic review and individual patient data meta-analysis. PLoS Med. 2017;14:e1002389. 28926573 10.1371/journal.pmed.1002389 PMC5604965

[39] Wall HK, Wright JS, Jackson SL, et al. How do we jump-start self-measured blood pressure monitoring in the United States? Addressing barriers beyond the published literature. Am J Hypertens. 2022;35:244–55. 35259238 10.1093/ajh/hpab170 PMC10061272

[40] West DM, Karsten J. Rural and Urban America Divided by Broadband Access, Commentary. Brookings Institution. Available at: https://www.brookings.edu/articles/rural-and-urban-america-divided-by-broadband-access/. Accessed 20 Nov 2023.

[41] Iyer S, Mehta P, Weith J, et al. Converting a geriatrics clinic to virtual visits during COVID-19: a case study. J Prim Care Community Health. 2021;12:21501327211000236. 33729044 10.1177/21501327211000235 PMC7975524

[42] Williams C, Shang D. Telehealth usage among low-income Racial and ethnic minority populations during the COVID-19 pandemic: retrospective observational study. J Med Internet Res. 2023;25:e43604. 37171848 10.2196/43604 PMC10185335

[43] Hill-Briggs F, Adler NE, Berkowitz SA, Chin MH, Gary-Webb TL, Navas-Acien A, et al. Social determinants of health and diabetes: a scientific review. Diabetes Care. 2020;44(1):258–79. 33139407 10.2337/dci20-0053 PMC7783927

[44] The White House. Broadband Equity Access Equity and Deployment (BEAD) program, Available at: https://internetforall.gov/program/broadband-equity-access-and-deployment-bead-program. Accessed 20 Nov 2023.

[45] Federal Communications Commission. Affordable Connectivity Program. Available at: https://www.fcc.gov/acp. Accessed 20 Nov 2023.

[46] The White House. The Build Back Better Framework. Available at: https://www.whitehouse.gov/build-back-better/. Accessed 20 Nov 2023.

[47] Shapero J. The Hill. Internet providers agree to continue discounted plans through end of 2024 as federal subsidies end. Available at: https://thehill.com/policy/technology/4695105-internet-providers-continue-discounted-plans-2024-federal-subsidies-end/. Accessed June 11 2024.

[48] Affordable Connectivity Program (ACP). Wind-Down Fact Sheet. Available at: https://www.fcc.gov/sites/default/files/ACP_Wind-down_Fact_Sheet_2024.pdf. Accessed 11 June 2024.

[49] Freeman S, Marston HR, Olynick J, et al. Intergenerational effects on the impacts of technology use in laterl: insights from an international, multi-site study. Int J Environ Res Public Health. 2020;17:5711. 32784651 10.3390/ijerph17165711 PMC7459619

[50] Pew Research Center. Internet/Broadband Fact Sheet. Available at: https://www.pewresearch.org/internet/fact-sheet/internet-broadband/. Accessed 20 November 2023.

